# Comparison of acetone and sodium phosphotungstic acid precipitation for sample enrichment prior to RT-QuIC for the detection of prion disease

**DOI:** 10.1186/s13104-026-07755-0

**Published:** 2026-03-19

**Authors:** Eric M. Nicholson, Susan E. Veneziano

**Affiliations:** United States Department of Agriculture Department of Agriculture, Agricultural Research Service, Virus and Prion Research Unit, National Animal Disease Center, Ames, IA 50010 USA

**Keywords:** Prion, TSE, Transmissible spongiform encephalopathy, CWD, Chronic wasting disease, RT-QuIC, Real time quaking induced conversion, Acetone, NaPTA, Sodium phosphotungstic acid

## Abstract

Transmissible spongiform encephalopathies, or prion diseases, are fatal neurodegenerative diseases that are medically, economically, and ecologically important. In livestock and wildlife, detection to prevent introduction into a new herd or to assess environmental contamination is imperative for disease management. Real time quaking induced conversion (RT-QuIC) is an amplification-based approach with higher sensitivity than traditionally used immunoassays for TSEs and has been used to detect the presence of disease associated prions from various sample types. RT-QuIC also pairs well with upstream sample enrichment methods. In this study we compare sample enrichment by sodium phosphotungstic acid (NaPTA) precipitation, to acetone precipitation. Acetone precipitation is widely used in preparation and analysis of proteins but less commonly used for prion disease samples. This work shows that for brain samples, which typically contain a high amount of disease associated prion protein, acetone and NaPTA provide similar results to unenriched samples. In fecal samples, which have a low abundance of disease associated prion protein, acetone enrichment results in greater sensitivity than either NaPTA or unenriched samples. Acetone enrichment can complement RT-QuIC affording laboratories a rapid sample enrichment approach to improve sensitivity of detection for low abundance prion samples collected from wild and farmed animals.

**Supplementary Information:**

The online version contains supplementary material available at 10.1186/s13104-026-07755-0.

## Introduction

Prion diseases, or transmissible spongiform encephalopathies (TSEs), include Creutzfeldt-Jakob disease (CJD) in humans, bovine spongiform encephalopathy (BSE) in cattle, chronic wasting disease (CWD) in cervids, and scrapie in sheep and goats. They are always fatal neurodegenerative disorders resulting from the misfolding of the cellular prion protein (PrP^C^) into its pathogenic form (PrP^Sc^). Misfolded PrP^Sc^ accumulates mainly in the central nervous system and, in CWD and scrapie, is also detected in lymphoid tissues, blood, urine and feces [3, 6, 28, 33].

The real-time quaking induced conversion (RT-QuIC) assay is a sensitive and specific in vitro assay designed to detect low levels of Prp^Sc^ in several different sample types including brain, lymphoid tissue, feces and blood [4, 5, 10, 13, 19, 29, 30]. Under specific assay conditions, PrP^Sc^ present in a sample (the seed) induces the conversion of monomeric recombinant PrP (rPrP, the substrate) into amyloid fibrils. RT-QuIC monitors the growth of PrP^Sc^ seeded fibrils in real-time using the amyloid binding dye thioflavin T (ThT). As amyloid fibrils from converted rPrP accumulate, fluorescence emitted from ThT binding increases [1, 2, 8, 21, 26, 27, 34]. Advantages of RT-QuIC are its sensitivity, as it has been demonstrated to detect prion disease in subclinical animals prior to detection by immunohistochemistry; high-throughput design; and suitability for testing antemortem samples, such as feces, that due to a low abundance of detectable PrP^Sc^ do not lend themselves to traditional TSE diagnostic methods [2, 4, 13, 18, 19]. However, due to the complex nature of biological samples, endogenous factors such as heme [7, 11] in PrP^Sc^ seeds have been found to interfere with the RT-QuIC reaction causing false negative or false positive results. In brain homogenate, high concentrations of polar lipids have also been demonstrated to inhibit RT-QuIC reactions and, in fecal extract, unidentified components, possibly bile acids or their derivatives, appear to interfere with the RT-QuIC reaction [4, 14]. These inhibitory factors are frequently overcome by diluting the seeds or performing an enrichment procedure to enhance the level of prions in the sample relative to other components.

Enrichment by precipitation removes inhibitory factors and can also be used to concentrate prions in samples that have lower levels of PrP^Sc^. Sodium phosphotungstic acid (NaPTA) precipitation is the commonly accepted enrichment procedure to purify and/or concentrate RT-QuIC seed samples. In brain homogenate, Levine et al., proposed that NaPTA works in conjunction with sarkosyl to precipitate PrP^Sc^ by separating the higher density PrP^Sc^ aggregates from neuronal lipids [20]. NaPTA precipitation is frequently used to enrich prions for a variety of downstream analyses. Enrichment by acetone precipitation is a commonly used method to nonspecifically separate proteins from other components in a solution to clean up or concentrate samples for use in downstream applications. It is a low-cost, simple procedure and can be easily applied to small sample volumes. The practical difference between acetone and NaPTA enrichment is that NaPTA is believed to interact specifically with PrP^Sc^, whereas acetone acts non-specifically by decreasing the solubility of most proteins in a sample while increasing the solubility of other components such as lipids and detergents. Thus, it is possible that if other proteins are inhibitory to the RT-QuIC reaction, acetone enriched samples would be less efficient at seeding RT-QuIC than NaPTA enriched samples. Acetone precipitation has also been shown to result in an approximately 1 log reduction in TSE infectivity using rodent models [29]. However, acetone has not been previously assessed for its effects on in vitro amplification. In this study, using scrapie infected sheep brain homogenate and fecal extract from white-tail deer infected with chronic wasting disease. In this proof of concept assessment of acetone precipitation as a cleanup for RT-QuIC, we first determined if acetone enrichment of PrP^Sc^ samples is compatible with the RT-QuIC assay and secondly, we compared acetone enriched PrP^Sc^ seed to NaPTA enriched PrP^Sc^ seed in the RT-QuIC reaction We used NaPTA and acetone enrichment as a cleanup step, and in all cases, samples were reconstituted in the initial volume following precipitation. This was done to directly compare enriched samples to unenriched samples.

## Methods

### Sample sources

Scrapie-infected and negative sheep brain homogenates prepared at 10% in 1X phosphate buffered saline (PBS) were obtained from previous studies [31]. All animal studies were carried out in accordance with the Guide for the Care and Use of Laboratory Animals (Institute of Laboratory Animal Resources, National Academy of Sciences, Washington, DC) and the Guide for the Care and Use of Agricultural Animals in Research and Teaching (Federation of Animal Science Societies, Champaign, IL); protocols were approved by the Institutional Animal Care and Use Committee at the National Animal Disease Center. Fecal samples from chronic wasting disease-infected and negative white-tail deer were provided by Dr. Tracy Nichols, Veterinary Services Cervid Health Program, United States Department of Agriculture, Animal and Plant Health Inspection Services. The samples were collected post-mortem from naturally infected animals in CWD positive herds as part of CWD surveillance. These animals are captive production animals and covered under United States federal law as it relates to chronic wasting disease control, specifically 9 cfr. 55.3 and 9 cfr. 81.

### Fecal sample preparation

Fecal samples were prepared as described [4, 5, 16]. Briefly, 10% extracts were prepared in fecal extraction buffer (20 mM Sodium Phosphate, pH 7.1, 130 mM NaCl, 0.05% Tween 20, 1 mM PMSF, and 1X Complete Protease Inhibitors (Roche) using a dissociator (GentleMACS, Miltenyi Biotec). After centrifugation at 18,000 x g for 5 min, supernatants were collected and stored at -80 °C.

### Protein enrichment

Equal volumes (0.1mL for both sample types) of 10% brain homogenate or fecal extract were subjected to either acetone or sodium phosphotungstic acid (NaPTA) precipitation [4, 16]. For acetone precipitation, 4 volumes of ice-cold acetone were added to 1 volume of homogenate, vortexed briefly and incubated for at least 1 h at -20 °C. Samples were then centrifuged at 15,800 x g for 15 min. The supernatant was removed and the pellets resuspended in a volume of 1X phosphate buffered saline equal to the starting volume of homogenate (0.1 mL).

For NaPTA precipitation, samples were incubated with sarkosyl in 10 mM Tris, pH 7.4 (2% final sarkosyl concentration) at 37 °C, 1400 rpm for 30 min in a Thermomixer (ThermoFisher). NaPTA in 170 mM MgCl_2_, pH 7.4 was added to the samples (0.3% final NaPTA concentration), and samples were further incubated at 37 °C, 400 rpm for 2 h in a Thermomixer. Samples were then centrifuged at 15,800 x g for 30 min. The supernatant was removed, resulting pellets were washed in NaPTA Wash Buffer (10 mM Tris, pH 7.4, 100 mM NaCl, 0.5% Triton-X 100 (v/v), 10 mM EDTA, 0.5% sodium deoxycholate (w/v) and 0.1% sarkosyl (w/v) and centrifuged at 15,800 x g for 15 min ([4]; [16]). The supernatant was removed and pellets were resuspended in a volume of 1X phosphate buffered saline equal to the starting volume of homogenate (0.1 mL).

### Recombinant protein expression and purification

*Escherichia coli* [BL21(l210] was transformed with the pET28a vector containing the bank vole PrP gene (amino acids 23–231 with methionine at 109; GenBank Accession AF367624) and recombinant bank vole prion protein was expressed and purified as previously described (Vrentas, Onstot, et al., 2012). The molecular weight and purity of the resulting protein was verified by SDS-PAGE and Coomassie Blue staining. Protein concentration was calculated from its absorbance at 280 nm using an extinction coefficient of 62,005 M^-1^cm^-1^ as calculated for bank vole prion protein [17].

### Real-time quaking induced conversion

Ten-fold dilutions of all samples were prepared in 1X PBS with 0.05% sodium dodecyl sulfate (SDS) for use as seeds in RT-QuIC reactions. The RT-QuIC reaction mixture was composed of 10 mM phosphate buffer, pH 7.0, 400 mM NaCl, 0.1 mg/mL recombinant bank vole prion protein, 10 µM thioflavin T (ThT), and 1 mM EDTA tetrasodium salt. Aliquots of reaction mixture (98 µL) were loaded into black walled, clear optic-bottomed 96-well plates (Nunc, ThermoFisher Scientific, USA). Reactions were seeded with 2 µL of prepared 10-fold dilutions of brain homogenate or fecal extract (0.001% final SDS concentration in reaction). Appropriate negative controls were included on every plate: unenriched, NaPTA enriched and acetone enriched negative sheep brain; or unenriched, NaPTA enriched and acetone enriched negative white-tail deer fecal extract. Plates were sealed with plate film and incubated at 42 °C (brain homogenate) or 37 °C (fecal extract) in a BMG FLUOstar Omega plate reader with cycles of 1 min shaking (700 rpm double orbital) and 1 min rest for 100 h. ThT fluorescence was measured every 15 min (bottom read, excitation 448 nm, emission 482 nm, manual gain 1400, 20 flashes per well, 0.2 s settling time) [22, 24].

Samples were run in quadruplicate and ThT fluorescence data is displayed as the average of four replicates for each time point for each sample. To be considered positive at least 2 replicates out the four must be positive [24]. The threshold for determining a positive sample was calculated as the mean value of ThT fluorescence for the first 3 h of 1 negative control sample plus 10 standard deviations from the mean. Time to threshold (lag time) was calculated by BMG MARS analysis software and presented as the average of 4 replicates per sample ([12, 22, 24]; [24]). Multiple samples and plates were run for each sample type. Representative results of each are shown here.

## Results

**Fig. 1:**
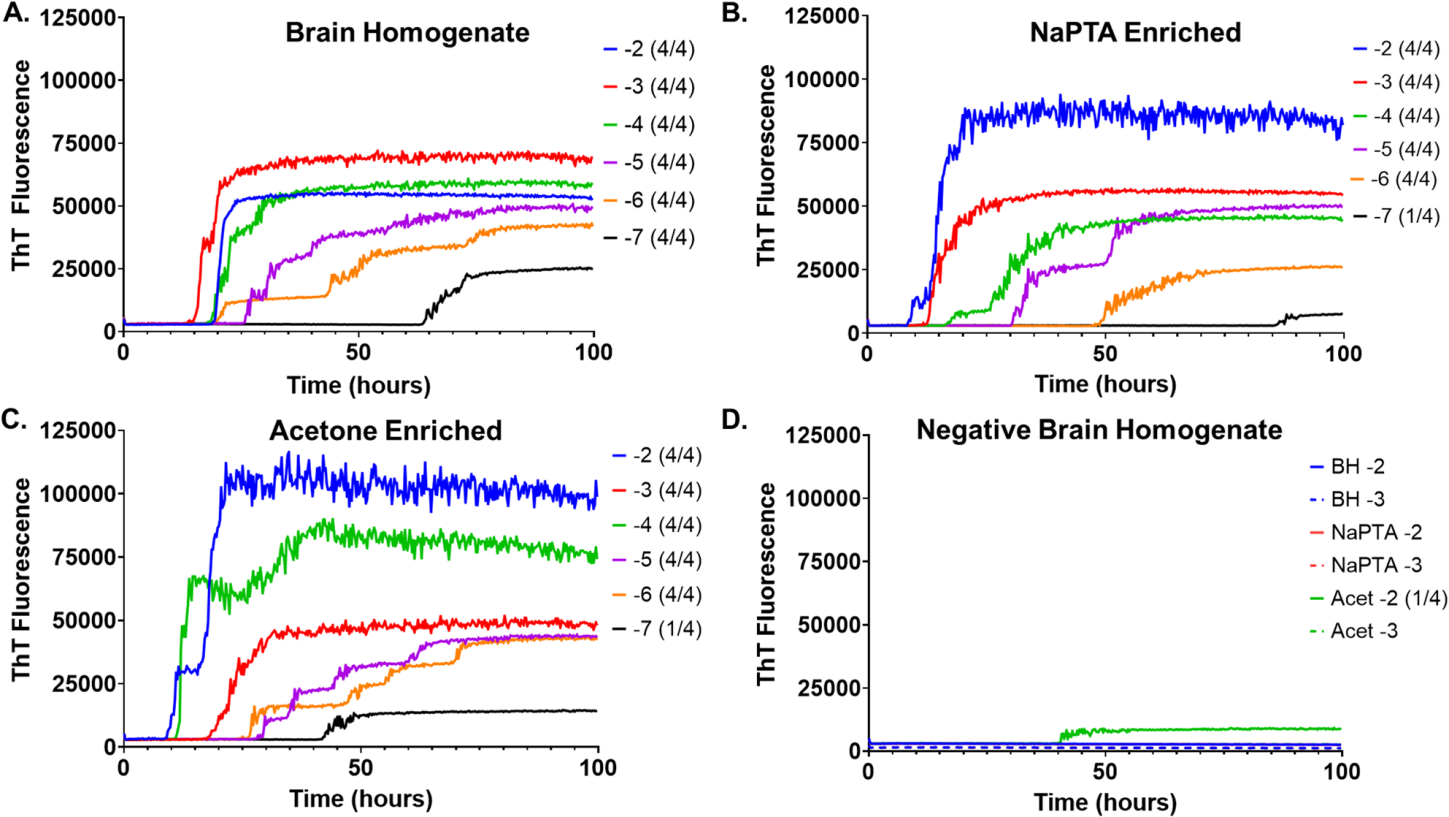
RT-QuIC results from **A**. scrapie-infected sheep brain homogenate; **B**. the same brain homogenate subjected to NaPTA precipitation; and (**C**) acetone precipitation. **D** RT-QuIC results from unenriched (BH), NaPTA precipitated (NaPTA), or acetone precipitated (Acet) negative sheep brain homogenate. Numbers in parenthesis indicate how many wells out of 4 replicates for this representative sample had ThT fluorescence exceed the threshold. At least 2 out of 4 replicates must exceed the threshold to meet the criteria for positivity

To determine if acetone is compatible with RT-QuIC and comparable to NaPTA, RT-QuIC reactions seeded by acetone enriched scrapie-brain homogenate or CWD fecal extract were compared to unenriched and NaPTA enriched scrapie brain homogenates or fecal extracts. Figure 1 represents RT-QuIC results comparing increasing dilutions of scrapie infected sheep brain homogenate seeds (1 A) to increasing dilutions of that same homogenate after NaPTA enrichment (1B) or acetone enrichment (1 C).

**Fig. 2:**
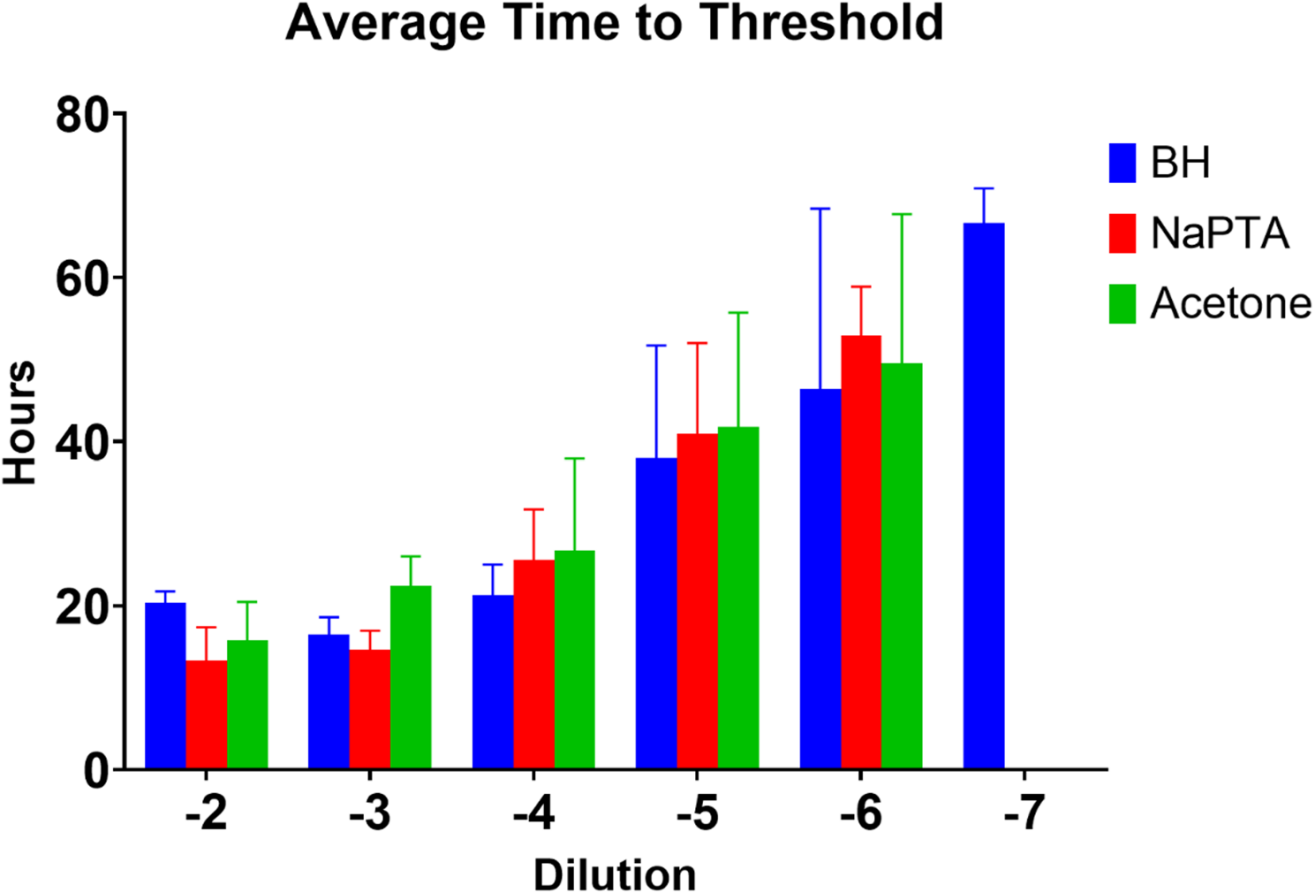
Average time to threshold of RT-QuIC for a representative scrapie-infected sheep brain homogenate (BH) versus NaPTA (NaPTA) and acetone enriched (Acetone). Time to threshold was calculated by MARS analysis software. Data represents the average of 4 replicates plus standard deviation

Figure 2 represents the average time to threshold for each sample at each dilution. The data demonstrate that acetone enrichment is compatible with RT-QuIC and is comparable to NaPTA in its ability to precipitate PrP^Sc^ that is able to seed RT-QuIC reactions. For brain homogenate, acetone enrichment did not appear to cause false positive RT-QuIC results as seen in the negative brain RT-QuIC results (Fig. 1D). While there did not appear to be a significant increase in time to threshold following enrichment by either NaPTA or acetone in positive samples when compared to unenriched brain homogenate (Fig. 2), the overall fluorescence signal was higher in the enriched samples (Fig. 1). In unenriched brain homogenate however, samples were positive out to the 10^− 7^ dilution but only out to the 10^− 6^ dilution for both the acetone and NaPTA enriched samples.

**Fig. 3:**
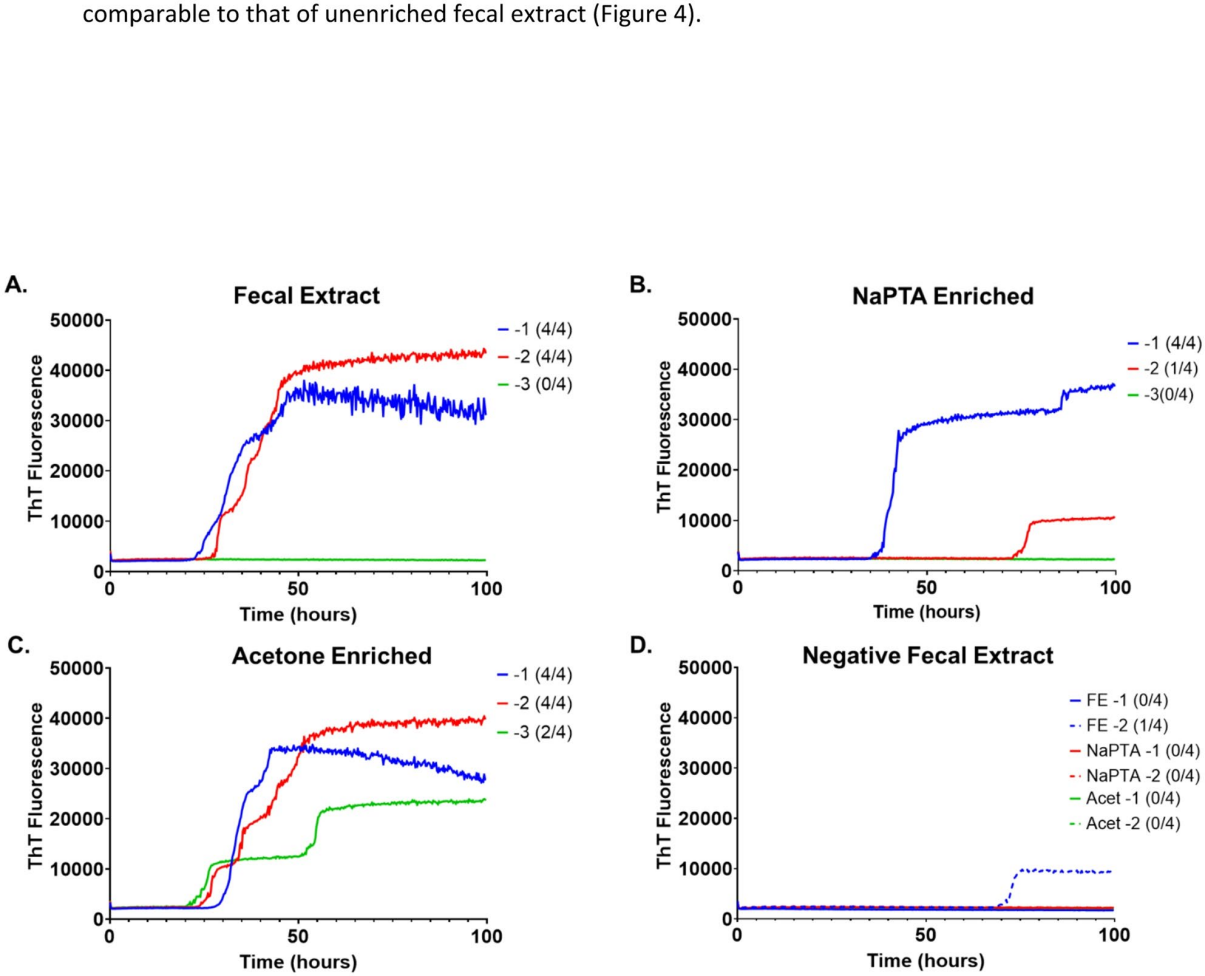
RT-QuIC Results from **A**. Fecal extract from CWD white-tail deer; **B**. the same fecal extract subjected to NaPTA precipitation; and (**C**) acetone precipitation. **D** RT-QuIC results from unenriched (FE), NaPTA precipitated (NaPTA), or acetone precipitated (Acet) fecal extract from known negative white-tail deer. Numbers in parenthesis indicate how many wells out of 4 replicates of the representative sample had ThT fluorescence exceed the threshold. At least 2 out of 4 replicates must exceed the threshold to meet the criteria for positivity

**Fig. 4:**
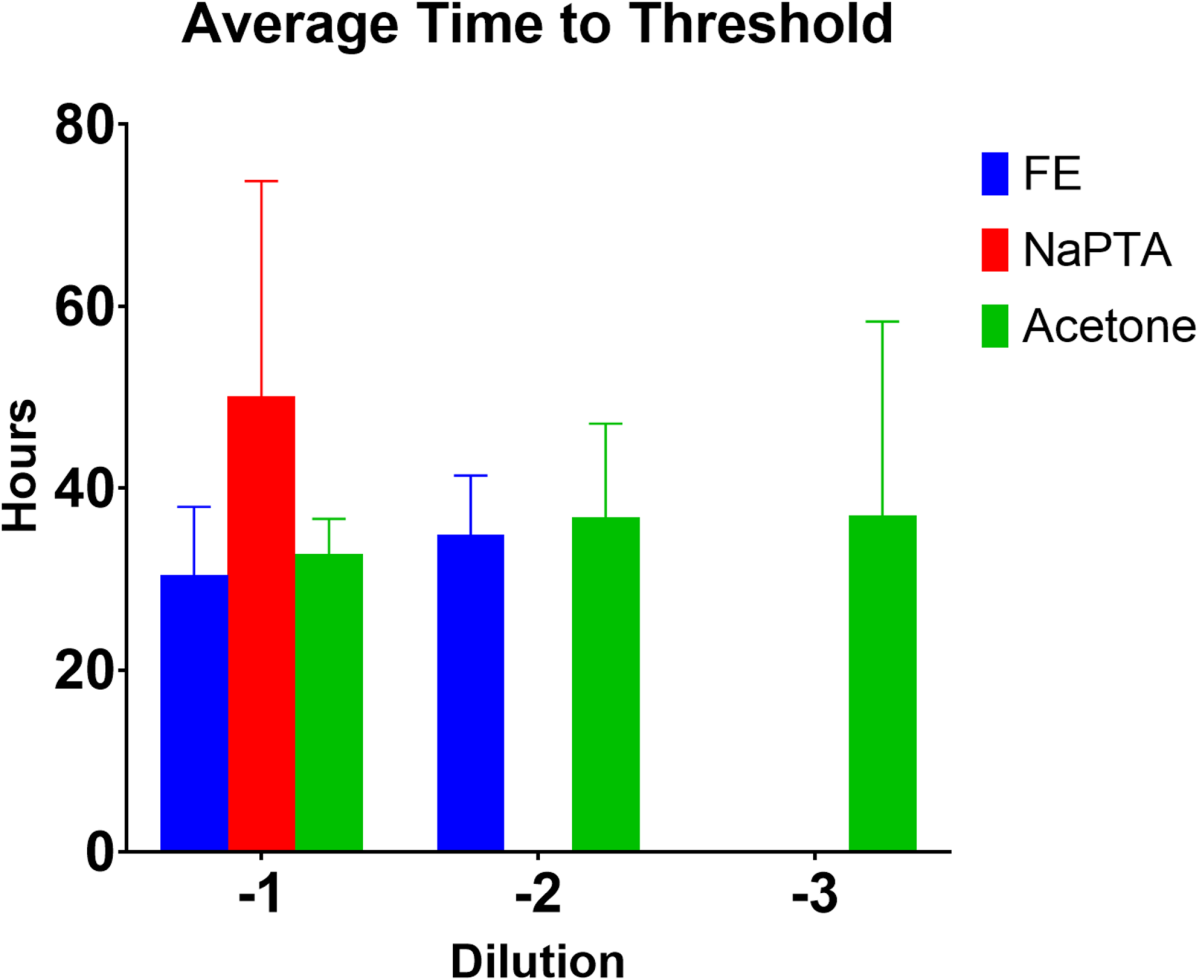
Average time to threshold of RT-QuIC for fecal extract from CWD infected white-tail deer (FE) versus NaPTA (NaPTA) and acetone (Acetone) enriched. Time to threshold was calculated by MARS analysis software. Data represents the average of 4 replicates plus standard deviation

Figures 3 and 4 represent RT-QuIC data from fecal extract of CWD infected white-tail deer collected in the field rather than an experimentally inoculated animal. Acetone enrichment of fecal extract, when used as a cleanup step, is compatible with the RT-QuIC reaction and did not appear to cause any false positive signals when used to enrich negative fecal extract (Fig. 3A, C and D). When compared to NaPTA enriched samples, acetone enrichment appears to improve the seeding activity of fecal extract as these samples were positive out to the 10^− 3^ dilution compared to only 10^− 1^ for NaPTA (Fig. 3B and C) and the average time to threshold was.

Under the conditions of this study, NaPTA enrichment of the fecal extract did not appear to improve the sensitivity or efficiency of the reaction. The NaPTA enriched samples were only positive at the 10^− 1^ dilution compared to the unenriched fecal extract, which was positive to the 10^− 2^ dilution (Fig. 3B compared to Fig. 3A). Also, the average time to threshold for the NaPTA enriched fecal extract was longer than that for unenriched fecal extract (Fig. 4).

## Discussion

In this proof of concept study two methods of enrichment paired to an amplification-based method for prion disease detection, RT-QuIC, were compared. This study shows that acetone, a non-specific protein precipitant, can be used for sample cleanup (enrichment) prior to RT-QuIC based detection of prion disease, and that it is as good as, if not better than NaPTA, a specific and commonly used PrP^Sc^ precipitant. For brain homogenate, the two enrichment methods provided comparable results with each other and with unenriched brain homogenate (Figs. 1 and 2). The total fluorescence was slightly higher in both enriched samples, but overall, the sensitivity was not increased relative to unenriched brain homogenate as demonstrated by the similar times to threshold (Fig. 2), and, at the highest dilution, only the unenriched brain homogenate was positive. In fecal extract, the acetone enriched sample and the unenriched sample exhibited comparable times to threshold, with the NaPTA enriched sample having the longest time to threshold. An increased time to threshold is suboptimal as it increases the overall required time needed for the assay. Only the acetone enriched fecal extract was positive at the 10^− 3^ dilution suggesting that acetone precipitation may increase sensitivity for fecal extracts, although a larger sample size than used for this proof-of-concept study would be needed to draw any firm conclusions. In all cases the samples were reconstituted in the initial volume following precipitation. This was specifically to afford the ability to compare the precipitated samples to each other and to the non-precipitated sample. It would be reasonable to reconstitute the samples in a lower volume resulting in more concentrated samples to evaluate in the RT-QuIC assay, but for the purposes of this study and comparison with unenriched samples, that was not done.

In this study, RT-QuIC did not detect fecal samples out to as high of dilutions as seen for brain homogenate and the total ThT fluorescence was lower in the fecal extract-seeded RT-QuIC reactions. This is likely due to lower amounts of PrP^Sc^ capable of seeding the RT-QuIC assay in fecal samples as well as the presence of more inhibitory material in fecal extract than in brain homogenate. Generally, both reduced seeding and the increased inhibitory material have led to the routine use of NaPTA enrichment when fecal samples are used for CWD detection [5, 16].

While acetone and NaPTA are roughly equivalent when utilized on brain homogenate, brain homogenate generally contains more PrP^Sc^ than other tissues in an infected host and enrichment is not generally used or needed. However, for fecal extract and other excretions and secretions, enrichment to remove inhibitory contaminants is desirable and a number of methods have been used including NaPTA [9, 15, 25]. Here we demonstrate that the use of acetone precipitation as enrichment for PrP^Sc^ prior to RT-QuIC as a viable alternative that performs as good as if not better than NaPTA precipitation.

## Limitations

The limitations of this study as presented here are sample size and the use of multiple species (sheep brain homogenate and deer feces). We routinely use acetone after having empirically observed the utility of the approach. For the purposes of this report we, however, selected disparate samples intentionally to examine the results with the intent that it inform the reader as to the limited gains for high abundance brain samples and the advantages for low abundance fecal samples.

## Supplementary Information

Below is the link to the electronic supplementary material.

Supplementary Material 1.

## Data Availability

The dataset used and/or analyzed during the current study is available from the corresponding author on reasonable request.
