# Supplementary material for: Comparison of acetone and sodium phosphotungstic acid precipitation for sample enrichment prior to RT-QuIC for the detection of prion disease
Source: BMC Res Notes. 2026 Mar 19;19:191. doi: 10.1186/s13104-026-07755-0 (PMC13122914; doi:10.1186/s13104-026-07755-0)
Supplement: Supplementary file 1 — Supplementary Material 1. [file 13104_2026_7755_MOESM1_ESM.docx]

**
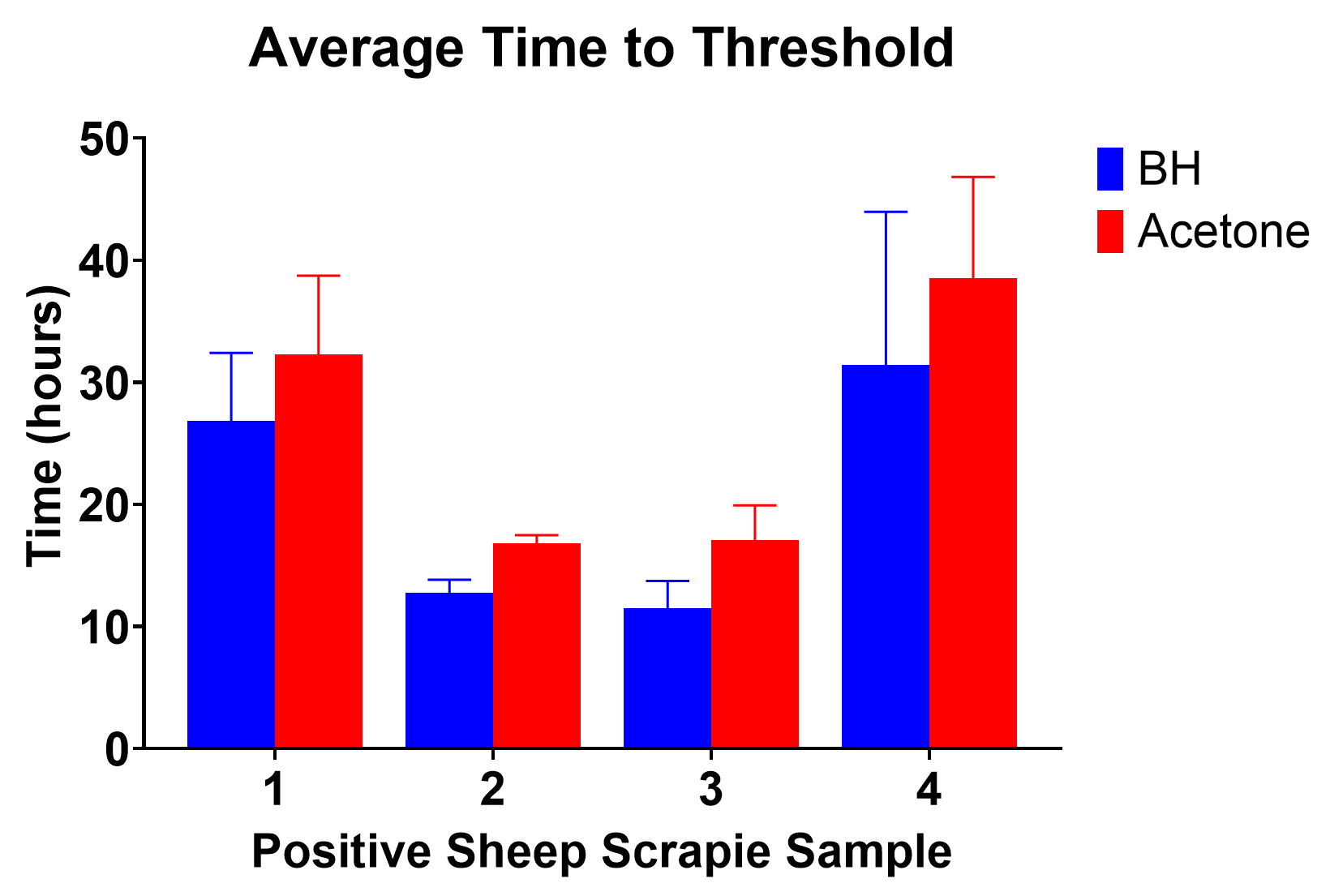
Supplementary Figures**

S1. Average time to threshold of RT-QuIC for various scrapie-infected sheep brain homogenate (BH) samples versus acetone enriched (Acetone). Time to threshold was calculated by MARS analysis software. Data represents the average of 4 replicates plus standard deviation.


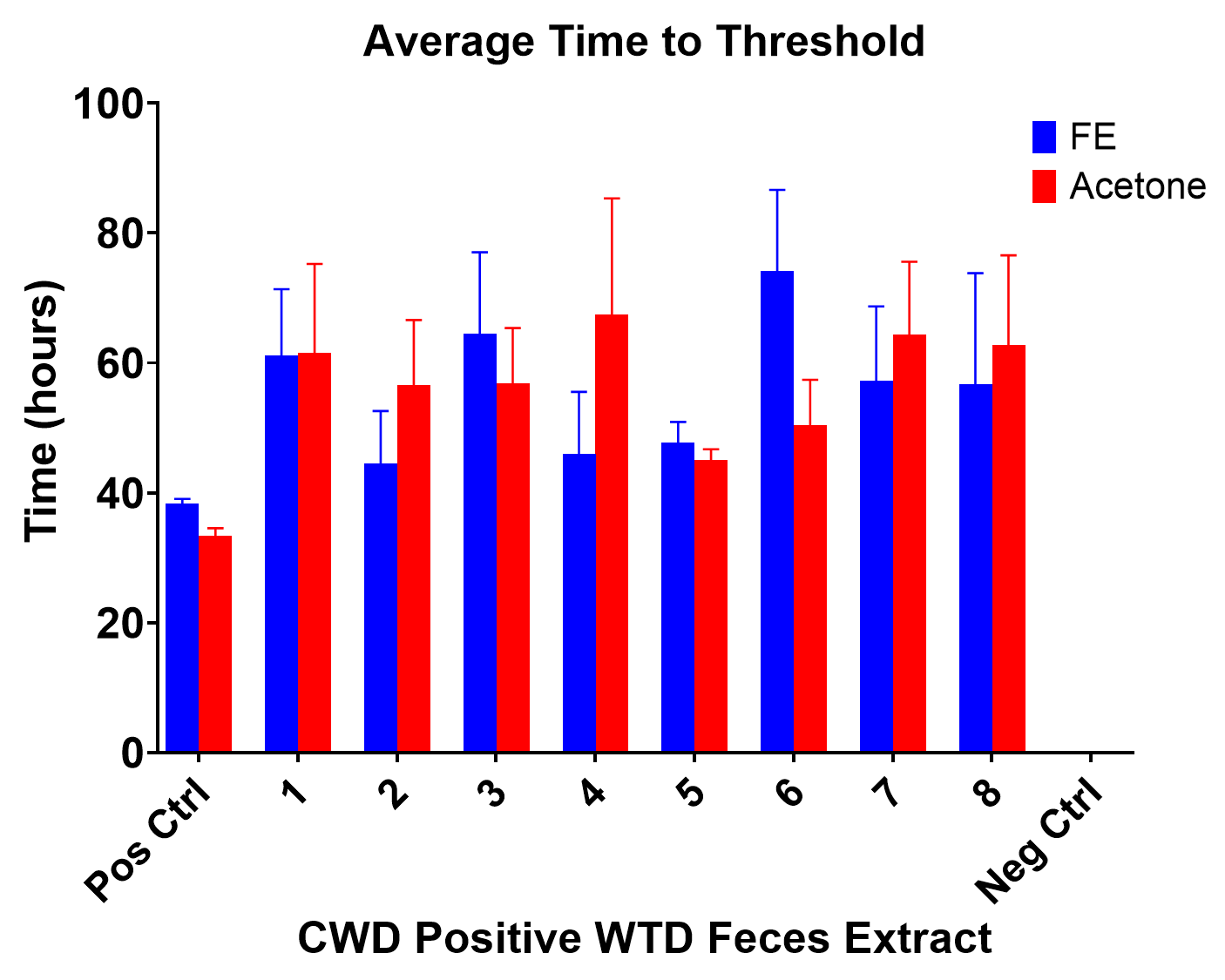
S2. Average time to threshold of RT-QuIC for various fecal extract from CWD infected white-tail deer (FE) versus acetone (Acetone) enriched. Time to threshold was calculated by MARS analysis software. Data represents the average of 4 replicates plus standard deviation.
